# Claudins in the Renal Collecting Duct

**DOI:** 10.3390/ijms21010221

**Published:** 2019-12-28

**Authors:** Janna Leiz, Kai M. Schmidt-Ott

**Affiliations:** 1 Department of Nephrology and Intensive Care Medicine, Charité-Universitätsmedizin Berlin, 12203 Berlin, Germany; janna.leiz@charite.de; 2 Molecular and Translational Kidney Research, Max-Delbrück-Center for Molecular Medicine in the Helmholtz Association (MDC), 13125 Berlin, Germany; 3 Berlin Institute of Health (BIH), 10178 Berlin, Germany

**Keywords:** epithelial barrier, barrier formation, collecting duct, tight junction, claudin

## Abstract

The renal collecting duct fine-tunes urinary composition, and thereby, coordinates key physiological processes, such as volume/blood pressure regulation, electrolyte-free water reabsorption, and acid-base homeostasis. The collecting duct epithelium is comprised of a tight epithelial barrier resulting in a strict separation of intraluminal urine and the interstitium. Tight junctions are key players in enforcing this barrier and in regulating paracellular transport of solutes across the epithelium. The features of tight junctions across different epithelia are strongly determined by their molecular composition. Claudins are particularly important structural components of tight junctions because they confer barrier and transport properties. In the collecting duct, a specific set of claudins (Cldn-3, Cldn-4, Cldn-7, Cldn-8) is expressed, and each of these claudins has been implicated in mediating aspects of the specific properties of its tight junction. The functional disruption of individual claudins or of the overall barrier function results in defects of blood pressure and water homeostasis. In this concise review, we provide an overview of the current knowledge on the role of the collecting duct epithelial barrier and of claudins in collecting duct function and pathophysiology.

## 1. Introduction

Selective barriers formed by epithelial monolayers are vital for many critical physiological processes [1]. These barriers are composed of intercellular multiprotein complexes along the apical-basal axis of epithelial cells creating direct cell-cell interactions and forming apical junctional complexes [2]. These complexes include tight junctions (TJs), adherens junctions, and desmosomes.

TJs are the most apical and diverse of these junctional complexes. They are formed by transmembrane as well as cytoplasmic proteins and are linked to the cytoskeleton. The protein composition of TJs varies greatly among different epithelia and determines the barrier properties and permeability [3]. 

Overall, TJs serve a dual function in epithelial layers acting as (a) a fence, separating membrane proteins between the apical and basolateral membrane and (b) as a gate by regulating size- and charge-selective movements of ions, solutes, and small molecules via the paracellular route [4,5,6,7]. Consequently, TJs are not only critical to establish and maintain cell adhesion and epithelial polarity but are also necessary to create selective paracellular permeability between compartments.

Several studies show that the main protein family mediating TJ characteristics and functions are the membrane-spanning claudins. So far, more than 25 different claudins have been identified, and at least 10 of them are expressed in spatiotemporal patterns along the renal nephron (for a detailed review see [8]).

Structurally, the transmembrane proteins connect in both *cis* and *trans* to neighboring claudins of the same, as well as the opposing, cell membranes forming TJ strands [9]. Claudins are sufficient to arrange those strands when expressed in cells lacking endogenous TJ formation [10].

Here, we review the role of claudins in epithelial barrier formation with emphasis on the renal collecting duct as well as their implications in collecting duct physiology and function.

## 2. The Renal Collecting Duct

The renal collecting duct is the most distal part of the renal tubules. It connects renal nephrons with the renal pelvis and—together with the distal convoluted tubule and the connecting tubule—it contributes to the aldosterone-sensitive distal nephron. In general, the renal collecting duct plays important roles in fine-tuning urinary composition, extracellular fluid volume, electrolyte balance, blood pressure regulation, water homeostasis, and acid-base regulation [11,12].

Although most of the water and solute reabsorption in the kidney occurs in the more upstream segments of the nephron, transport variability in the renal collecting duct is significantly higher, and ion and water transport are under strict hormonal control [11]. This permits to adjust reabsorption and secretion to prevalent physiological conditions and to control the body’s water and electrolyte balance closely.

**Figure 1:**
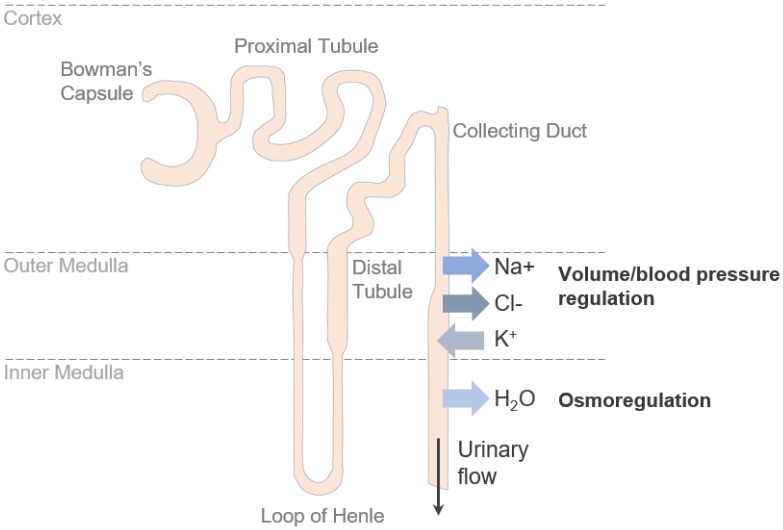
Transport in the renal collecting duct. In the renal collecting duct Na+ and Cl− are reabsorbed, while K+ is secreted into the urine. A steep osmolality gradient between the interstitium and lumen across the epithelial barrier is the driving force for water reabsorption.

The main solutes reabsorbed in the collecting duct are sodium (Na+) and chloride (Cl−), while potassium (K+) is secreted into the urine [13] (Figure 1). Transport of these ions occurs via the transcellular route—mediated by channels and membrane transporters—and via the paracellular route—mediated by TJs.

## 3. Claudins and Their Roles in Collecting Duct Epithelial Barrier Formation and Paracellular Ion Transport

In general, claudins can be pore- or barrier-forming [14]. As in other segments of the renal nephron, it is believed that the expressed claudins are the main mediators of TJ permeability characteristics, and thus, control the paracellular pathway in the collecting duct [9,15]. Among the predominately expressed claudins in the renal collecting duct are claudin-3 (Cldn-3), -4 (Cldn-4), -7 (Cldn-7) and -8 (Cldn-8) [16,17]. Furthermore, a recently published study proposes a role for claudin-19 (Cldn-19) in the renal collecting duct [18]. Overall, the present knowledge strongly supports the theory that these claudins either enforce the epithelial barrier in general or build a paracellular Cl− channel, while other aspects of their functions are less clear (Table 1). 

In the following sections, we will briefly review the published literature on the functions of these claudins in the collecting duct. As a general note, it should be acknowledged that gain-of-function studies utilizing overexpression of claudins in cell lines have a general limitation due to the influence of the endogenous components of the TJ in each cell line. The expressed claudins in the respective chosen model make it difficult to determine the exact impact of single claudins on permeability characteristics. In contrast, loss-of-function studies, if carried out in the appropriate system (i.e., the collecting duct in vivo or cell lines that accurately represent collecting duct epithelia), are more appropriate to outline the molecular functions of each claudin. 

### 3.1. Cldn-3

Several studies have found that Cldn-3 enforces the paracellular barrier [19,20]. Its overexpression in otherwise leaky Madin-Darby Canine Kidney (MDCK) II cells leads to an altered TJ structure with increased transepithelial resistance (TER) and decreased permeability for ions as well as molecules of 332 Da and 4 kDa, indicating a general role for Cldn-3 in barrier formation and enforcement [19]. Furthermore, Cldn-3 has been described to promote tubule formation in vitro and might thus play a role during tubule morphogenesis in the developing kidney [30]. The conditional knockout of Cldn-3 resulted in increased urinary pH without any effect on urine or plasma electrolytes [21]. However, the collecting duct-specific role of Cldn-3 has not been studied so far.

### 3.2. Cldn-7

Cldn-7-deficient mice die shortly after birth due to severe renal salt wasting and dehydration [27]. Interestingly, collecting duct cells isolated from Cldn-7-deficient mice demonstrate an increase in TER and show decreased paracellular permeability for both Na+ and Cl− [31]. This suggests that Cldn-7 may form a non-selective paracellular channel facilitating Cl− and Na+ reabsorption in collecting ducts. 

Different and partially conflicting roles of Cldn-7 have been suggested in other experimental systems, complicating the interpretation of Cldn-7 biology. Overexpression of Cldn-7 in LLC-PK1 cells induces an increase in TER accompanied by a reduced Cl− and elevated Na+ conductance [24]. In contrast, the knockdown of Cldn-7 in those cells leads to loss of anion selectivity and decreased Cl− permeation, whereas in cation-selective MDCK cells, knockdown of Cldn-7 leads to increased Na+ permeability [25]. Mutation experiments in vitro demonstrated the importance of specific negatively charged amino acids in the extracellular loop of Cldn-7 for paracellular permeability and charge selectivity [32]. These contrasting results could indicate different roles for Cldn-7, depending on the cellular background, and endogenously expressed TJ components.

### 3.3. Cldn-4 and Cldn-8

Both, Cldn-4 and Cldn-8, are thought to serve as a cation barrier [33,34,35] and an anion channel [22]. This hypothesis is supported by data from in vivo experiments. Collecting duct-specific knockout of either Cldn-4 or Cldn-8, causes hypotension, hypochloremia, metabolic alkalosis and renal wasting of Na+ and Cl− [23,29]. These phenotypes would be consistent with Cldn-4 and Cldn-8 acting as paracellular Cl− channels, which are necessary for a paracellular “chloride shunt” required for effective transcellular Na+ reabsorption (via the epithelial sodium channel).

Different effects of Cldn-4 overexpression on charge selectivity, depending on the used cell model, have been described. Overexpression of Cldn-4 leads to an increase in TER in cation-selective MDCK II and anion-selective LLC-PK1 cells, but a decrease in Na+ permeability is only observed in MDCK II cells [34]. 

Cldn-8 has been shown to be necessary to recruit Cldn-4 to the TJ and to implement the protein into the junctional complex. In the absence of Cldn-8, Cldn-4 is mainly found in the endoplasmic reticulum and the Golgi complex, but not in the apical cell membrane where TJs are located [22]. Thus, Cldn-8 knockout causes a functional double knockout on the TJ level due to its requirement for correct Cldn-4 localization [22]. 

### 3.4. Cldn-19

In addition to the longer known claudins of the renal collecting duct described above, a recently published study indicates a role for Cldn-19, which had previously been linked to thick ascending limb functions [36,37,38]. Cldn-19 is associated with tightness and cation selectivity of the epithelial barrier. Interestingly, the TJ localization of Cldn-19 is promoted by a low osmolality, whereas high osmolality favors an intracellular localization, suggesting that it may contribute to tonicity-induced changes in paracellular ion selectivity [18]. This role of a claudin in epithelial adaption to the changing osmolality along the corticomedullary axis provides an interesting aspect of TJ physiology but collecting duct-specific knockout models of Cldn-19 have not yet been generated.

## 4. The Collecting Duct Epithelial Barrier in Electrolyte-Free Water Reabsorption

Although water transport in the renal collecting duct is not directly facilitated by water channel-forming claudins (contrasting with observations of Cldn-2 in the proximal tubule [39]), TJs in the collecting duct contribute to water reabsorption indirectly.

The driving force for water reabsorption in the renal collecting duct is a steep osmolality gradient formed by high concentrations of osmolytes in the interstitium, which increases towards the renal medulla [40]. The tight collecting duct epithelial barrier is crucial to maintain the osmolality gradient between the tubular lumen and the interstitium.

Water transport in the renal collecting duct is mainly controlled by arginine vasopressin (AVP), also called antidiuretic hormone. If the water content in the body is low, AVP binds to its type 2 receptor (V2R) localized in the basal cell membrane of collecting duct principal cells and stimulates the expression of aquaporin-2 (AQP2) water channels. Furthermore, AVP triggers a signaling cascade leading to the accumulation of AQP2 in the apical membrane (for a detailed review see [11,41]). This mechanism enables reabsorption of electrolyte-free water from the intraluminal urine and forms the basis of urinary concentrating ability. Inactivation of either V2R or AQP2 leads to polyuria with massive excretion of electrolyte-free water, a condition called nephrogenic diabetes insipidus [42].

A recent study has demonstrated the importance of an intact epithelial barrier in the renal collecting duct for efficient water reabsorption [43]. Deletion of the transcriptional regulator Grainyhead-like 2 (Grhl2), an epithelial transcription factor that induces the expression of barrier-enforcing molecular TJ components including Cldn-4 [44], results in a leaky collecting duct epithelium and a decreased TER across the collecting duct epithelium. Leakage of interstitial osmolytes across the Grhl2-deficient collecting duct epithelium is associated with defective retention of osmolytes in the interstitium of the inner medulla. Grhl2-deficient mice show signs of diabetes insipidus and fail to concentrate their urine adequately, although AQP-mediated water transport across the apical and basolateral membranes of Grhl2-deficient collecting ducts is intact [43]. This indicates that a tight collecting duct epithelial barrier is crucial for the maintenance of osmolality gradients and for effective collecting duct water reabsorption. Interestingly, Grhl2 deficiency (unlike deficiencies of individual claudins) was not associated with abnormalities of Cl− and Na+ reabsorption. It needs to be acknowledged that Grhl2 functions are not restricted to the effects on the TJ, which may explain the difference in phenotypes. In addition, it is possible that the barrier defect of Grhl2-deficient collecting duct cells is more profound, leading to non-ion-selective leakage of interstitial osmolytes into hypotonic urine.

## 5. Aldosterone and Its Role in Transcellular and Paracellular Transport Regulation

In the renal collecting duct, Na+ transport is separated from Cl− transport [45]. Na+ reabsorption from the intraluminal urine occurs transcellularly via the epithelial sodium channel (ENaC) that locates to the apical membrane of collecting duct principal cells. This generates a lumen-negative potential, providing a driving force for K+ secretion via the renal outer medullary potassium channel (ROMK). In contrast, Cl− reabsorption in the collecting duct occurs predominantly via the paracellular route. This “chloride shunt” is important to limit the built-up of a lumen-negative potential and, thereby, facilitates continued Na+ reabsorption and prevents excessive K+ secretion. 

The key regulator of ENaC is aldosterone, a hormone secreted from the adrenal gland in response to hyperkalemia and hypovolemia [11,46]. Overall, aldosterone plays a central role in blood pressure regulation by controlling plasma Na+ and K+ levels and thus indirectly influences water retention or loss. However, growing evidence supports the hypothesis that aldosterone controls Na+ reabsorption and K+ secretion not only by regulating the abundance of ENaC and increasing transcellular transport but also by adjusting paracellular Cl− permeability in multiple ways:

For instance, aldosterone triggers the expression of Cldn-8 when ENaC is active, presumably to seal the paracellular route for Na+ back flux. Thereby net flux of Na+ can be increased [28].

**Figure 2:**
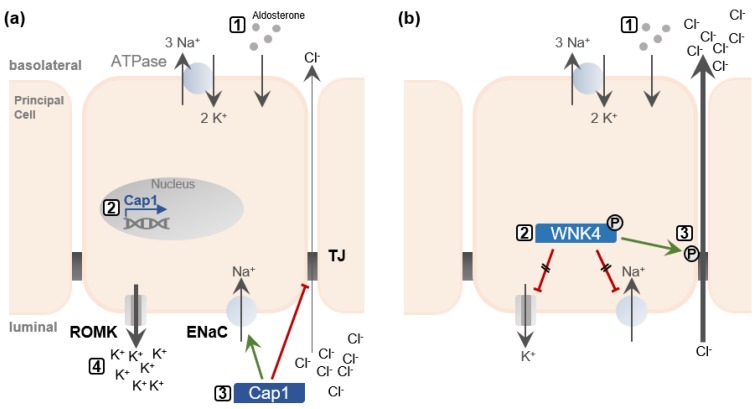
(**a**) Aldosterone-mediated induction of channel-activating protease 1 (Cap1) activates Na+ reabsorption, but simultaneously inhibits the paracellular “chloride shunt”, resulting in excessive K+ excretion. When aldosterone is secreted (1), it triggers the expression of Cap1 in principal cells of the renal collecting duct (2). Cap1 inhibits Cl− reabsorption directly by disrupting *trans*-interactions of Cldn-4. Simultaneously, Cap1 activates apical Na+ channels (ENaC), and thus, increases the transcellular reabsorption of Na+ (3). Consequently, the growing luminal negative potential drives K+ secretion into the urine via renal outer medullary potassium (ROMK) channels (4). (**b**) Aldosterone activates ENaC, ROMK, and the paracellular “chloride shunt” via with no lysine kinases 4 (WNK4) phosphorylation. When aldosterone is secreted (1), it leads to the phosphorylation of WNK4. This suspends the tonic inhibition of ROMK and ENaC by WNK4 (2). Furthermore, WNK4 phosphorylates claudins located to the tight junctions (TJs) of the renal collecting duct and thereby increases Cl− reabsorption (3). Green arrows indicate positive regulation, red lines indicate inhibition.

Additionally, aldosterone regulates channel-activating protease 1 (Cap1) [47]. Cap1, in turn, stimulates ENaC and inhibits the Cl− conductivity by directly regulating Cldn-4 *trans*-interactions [23,48]. Thereby K+ secretion is favored over Cl− reabsorption (Figure 2a).

Aldosterone also induces the phosphorylation and activation of the with no lysine kinases 4 (WNK4) [49]. Expression of WNK4 in MDCK II cells has been shown to reduce the TER and increase Cl− permeability, without affecting TJ structure [50]. Activated WNK4 phosphorylates Cldn-4 on threonine residues decreasing the cells’ TER and increasing apical to basal anion passage [51].

In the absence of aldosterone, WNK4 inhibits ENaC and ROMK activity and thus directly opposes Cap1 [52,53]. WNK4 phosphorylation suspends this inhibition (Figure 2b). Taken together, this indicates the possibility that aldosterone might regulate claudins through Cap1 and WNK4, coordinating Cl− reabsorption or K+ secretion, respectively.

Kahle and colleagues proposed WNK4 as the functional switch regulating Na+ and Cl− reabsorption independently from K+ secretion depending on the physiological conditions [53]. However, the factors facilitating different functional states of WNK4 haven’t been provided, and to our knowledge, it is still unknown how the reverse actions of Cap1 and WNK4, that are both mediated by aldosterone, are regulated to decide in favor of K+ secretion or Cl− reabsorption, respectively.

In diabetes, the role of aberrant aldosterone signaling in the progression of renal disease has long been established [54,55]. Mediated by the divergent aldosterone levels, Cldn-4 and Cldn-8 are overexpressed in the distal nephron from type 1 diabetic rats, and the expression of WNK4 and its co-localization with Cldn-4 and Cldn-8 is also increased [56,57]. This might result in increased activation of Cldn-4 and Cldn-8 by WNK4 under diabetic conditions and could implicate disturbed paracellular transport in renal disease progression. However, additional experimental evidence verifying this hypothesis is needed.

## 6. Chloride Reabsorption in Renal Collecting Ducts and Potential Involvement in Disease

**Figure 3:**
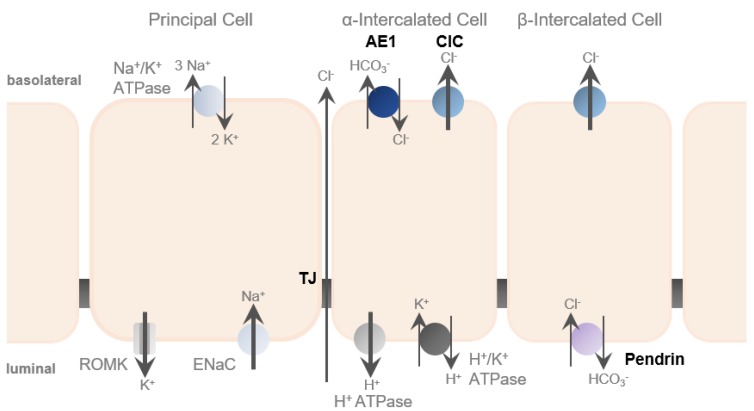
Chloride transport in the renal collecting duct. Chloride is transported via the paracellular route mediated by tight junctions (TJs), as well as, via the transcellular route using transporters and channels in α- and β-intercalated cells (depicted in bold).

The aldosterone-sensitive distal nephron is the main side of Cl− reabsorption. It occurs via multiple ways, including paracellular transport via TJs as well as transcellular pathways in intercalated cells [12,23,29] and is driven by the lumen-negative transepithelial potential generated by the unilateral Na+ reabsorption to maintain electroneutrality (Figure 3). As in aldosterone signaling, it becomes increasingly evident that crosstalk between paracellular and transcellular transport occurs.

The importance of efficient Cl− reabsorption becomes obvious in claudin-deficient mouse models. For example, Cldn-7 deficiency in mice is lethal within 12 days after birth due to severe salt-wasting and subsequent chronic dehydration (see above) [27]. Cldn-7^−/−^ mice show reduced ROMK and increased ENaC, Aqp2, and Na+ Cl− cotransporter mRNA. These changes in channel expressions are probably due to a compensatory mechanism to inhibit further urinary loss of salt and water [27]. 

It has long been established that accumulation of luminal Cl− depolarizes the membrane, and thereby, inhibits the apical Na+ channel ENaC [58]. Several studies also have demonstrated that pathological increases in Cl− reabsorption are associated with diseases, such as pseudo-hypoaldosteronism type II (PHA-II) or Gordon’s syndrome.

PHA-II is a rare Mendelian syndrome leading to hypertension, hyperkalemia, and metabolic acidosis [59]. These symptoms are the exact opposite of the Cldn-4 and Cldn-8 knockout phenotypes in mice [23,29]. 

Interestingly, a long-known causative gene for PHA-II is WNK4 [60], and WNK4 can regulate Cl− conductance presumably by phosphorylation of Cldn-4 and Cldn-8 [50]. The PHA-II-causing mutation of WNK4 increases paracellular Cl− permeability in vitro [49,50]. Consistent with the proposed Cl− pore activity of Cldn-4 and Cldn-8, this is an additional indicator of an activating effect of claudin phosphorylation by WNK4. Furthermore, a recent study demonstrates that WNK4 is overexpressed in Cldn-7-deficient cultured, collecting duct cells [31]. Conversely, the PHA-II mutant of WNK4 is associated with increased Cldn-7 phosphorylation and enhanced paracellular Cl− conductivity [26]. This adds a new aspect and raises the possibility that claudins might act both up- and down-stream of WNK4 to regulate paracellular transport.

Another gene associated with PHA-II is KLHL3, encoding Kelch-like protein 3 [61,62]. Interestingly, KLHL3 normally induces ubiquitination and degradation of Cldn-8, while disease-associated mutations of KLHL3 abolish the interaction of KLHL3 with Cldn-8 [29]. In addition, KLHL3 leads to the ubiquitination of WNK4. In line with PHA-II symptoms, loss of KLHL3 increases Cl- permeability in vivo [29], which may contribute to the disease phenotype.

## 7. Conclusions

Collecting duct epithelia express claudins enforcing a high TER such as Cldn-3, -4, -7, -8, and -19, consistent with a demand for strong epithelial barrier function in the presence of steep transepithelial gradients. Based on our current knowledge, these claudins are thought to either support a tight barrier in general or to act as a cation barrier and/or an anion channel. 

Consequently, the renal collecting duct is comprised of an especially tight epithelial barrier compared to other, more upstream segments of the nephron. As a result, the collecting duct lumen and interstitium are strictly separated. Nevertheless, the TJs of the collecting duct exhibit a regulated paracellular permeability for ions such as Cl-. Hence, the collecting duct TJ participates in a range of physiological functions: 

It allows for controlled paracellular transport of Cl- to the interstitium, which is important in the setting of Na+ reabsorption via ENaC and secretion of K+ via ROMK.

It prevents paracellular diffusion and back flux of osmolytes into the urine and thus promotes the formation of steep gradients across the epithelium, which are necessary to drive electrolyte-free water reabsorption. 

Future research will be needed to elucidate further the precise mechanisms that regulate TJ properties and that mediate crosstalk between paracellular and transcellular transport processes and how these processes relate to renal pathophysiology.

## Figures and Tables

**Table 1:** Claudins expressed in the renal collecting duct and their proposed function in barrier formation and collecting duct tight junctions.

Claudin	Proposed Function in the Collecting Duct Tight Junction	Knockout Phenotype In Vivo
Cldn-3	General barrier function [19,20]	Increase in urinary pH, no electrolyte abnormalities [21]
Cldn-4	Cl− channel, cation barrier [22]	Hypotension, hypochloremia, metabolic alkalosis, renal wasting of Na+ and Cl− [23]
Cldn-7	Cl− and Na+ channel [24,25,26,27]	Severe renal salt wasting, dehydration [27]
Cldn-8	Cl− channel, cation barrier [28,29]	Hypotension, hypochloremia, metabolic alkalosis, renal wasting of Na+ and Cl− [29]
Cldn-19	Adaption of barrier selectivity to osmolality [18]	-

## Abbreviations

AQP	Aquaporin
AVP	Arginine vasopressin
Cap1	Channel-activating protease 1
Cldn	Claudin
Cl-	Chloride
ENaC	Epithelial sodium channel
Grhl2	Grainyhead-like 2
K+	Potassium
KLHL3	Kelch-like protein 3
MDCK	Madin-Darby Canine Kidney
mIMCD3	Mouse inner medullary collecting duct
Na+	Sodium
NCC	Na+ Cl− cotransporter
PHA-II	Pseudohypoaldosteronism type II (also known as Gordon’s syndrome)
ROMK	Renal outer medullary potassium channel
TER	Transepithelial resistance
TJ	Tight junction
V2R	Vasopressin type 2 receptor
WNK4	With no lysine kinases 4
